# Aer is a bidirectional redox sensor mediating negative chemotaxis to antibiotic-induced ROS in *Escherichia coli*

**DOI:** 10.1128/mbio.00381-26

**Published:** 2026-03-23

**Authors:** Nabin Bhattarai, Jeremy P. Moore, Shelley Payne, Rasika M. Harshey

**Affiliations:** 1 Department of Molecular Biosciences and LaMontagne Center for Infectious Diseases, The University of Texas at Austin 196204, Austin, Texas, USA; 2 Molecular, Cellular and Developmental Biology, Yale University 118551, New Haven, Connecticut, USA; The Ohio State University, Columbus, Ohio, USA

**Keywords:** Aer chemoreceptor, aerotaxis, redox sensing, ROS, negative chemotaxis

## Abstract

Bacterial swarms employ many mechanisms to reduce their exposure to antibiotics, including actively avoiding them. We show in this study that the avoidance response in *Escherichia coli* swarms is dependent on the chemoreceptor Aer, known to perform oxygen- or aero-taxis by sensing metabolic flux through the electron transport chain (ETC) via a flavin adenine dinucleotide (FAD) cofactor bound to its Per-Arnt-Sim (PAS) domain. We find that Aer generates an FAD-dependent repellent or negative chemotaxis signal to the known reactive oxygen species (ROS) H₂O₂, thus sensing oxidative stress. Expression of the catalase–peroxidase enzyme KatG, which scavenges intracellular ROS, abrogates the repellent signal generated by both H₂O₂ and antibiotics. The ability of Aer to detect both oxidizing and reducing intracellular environments redefines its functional range and establishes Aer as a bidirectional redox sensor. Our study also provides independent behavioral evidence that certain antibiotics generate intracellular ROS, while offering a sensitive assay for detecting these reactive species.

**IMPORTANCE:**

Motile bacteria rely on aerotaxis to seek environments that maximize energy production. We show that in *Escherichia coli*, Aer mediates not only positive chemotaxis toward favorable redox conditions but also negative chemotaxis in response to unfavorable ones, actively moving away from oxidizing environments, including reactive oxygen species (ROS)-generating antibiotics, a previously unrecognized behavioral mechanism for bacterial survival under oxidative stress. The ability of Aer to detect both oxidizing and reducing cellular environments reveals an unexpected sensory versatility, shared to varying degrees by other *E. coli* chemoreceptors.

## INTRODUCTION

Flagella-driven movement is used by bacteria to explore their surroundings, locate favorable environments, and avoid harmful ones (1, 2). Flagella enable bacteria to move in two distinct ways: individually through liquid environments, known as swimming, or collectively across semi-solid surfaces, known as swarming. Resistance to antibiotics—both adaptive and genetic—has been observed during both swimming in spatially complex environments (3, 4) and during swarming (5–7). High cell densities have been seen to contribute to such resistance during both swimming (8) and swarming (9). When exposed to sublethal concentrations of antibiotics, many motile bacterial species exhibit altered motility patterns (10–12), actively moving away from regions of high drug concentration (13). We show in this study that active avoidance of certain antibiotics is controlled by the chemotaxis system in *Escherichia coli*.

The chemotaxis pathway of *E. coli* allows the bacteria to sense attractants and repellents and navigate its surroundings with the help of 4–6 peritrichous flagella, driven at their base by a bidirectional rotary motor powered by proton motive force (PMF) (14–16). The chemotaxis machinery biases flagellar rotation in response to sensory inputs, translating environmental cues into directed changes in swimming behavior. Chemoreceptors, also called methyl-accepting chemotaxis proteins (MCP), sense specific ligands in the periplasm and convey this information to a linked cytoplasmic kinase CheA (17). Attractants and repellents change MCP conformation to either inhibit or activate CheA autophosphorylation (CheA-OFF or ON, respectively). CheA-ON phosphorylates two response regulators—CheY and CheB. CheY~P interacts with the bidirectional flagellar motor to change its default counterclockwise (CCW) rotation direction to clockwise (CW) (15). Phosphatase CheZ dephosphorylates CheY~P to terminate the activation signal, restoring CCW rotation. The directionality of motor rotation, therefore, reports on CheA activity. Every signaling event is followed by an adaptation event that resets the pre-signaling conformation of the MCPs. This is achieved by CheB and CheR. CheB~P is a methylesterase, which preferentially interacts with the CheA-ON state of receptors and shifts them toward the OFF state, while CheR is a methyltransferase that preferentially interacts with the CheA-OFF state of the receptors and methylates E residues in the MCPs to return them to a CheA-ON state (17).

**Fig 1:**
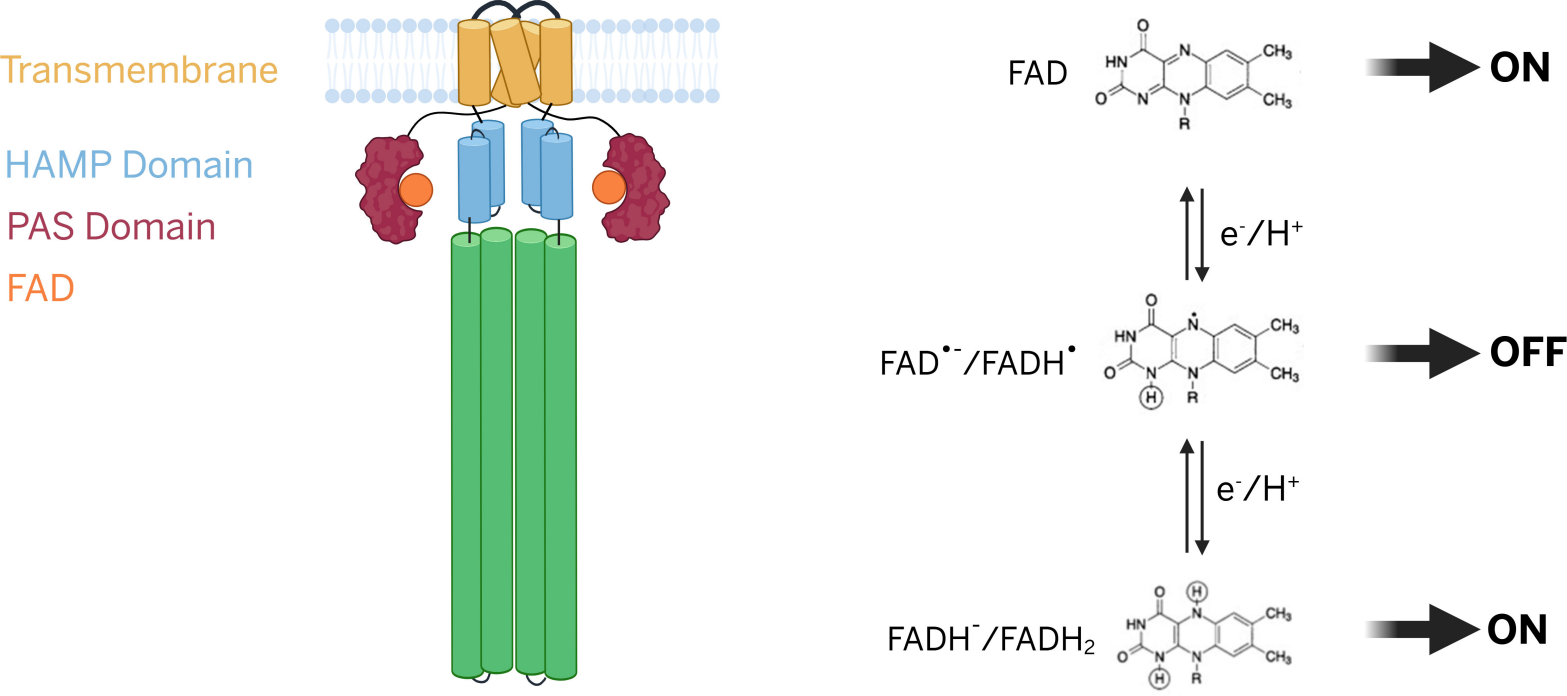
Model of Aer-mediated redox signaling and kinase regulation. Schematic representation of the Aer dimer showing the organization of signaling domains: HAMP (blue), PAS (burgundy), and bound FAD cofactor (orange). The redox state of FAD bound within the PAS domain determines Aer signaling output. Fully oxidized FAD maintains the kinase in the ON state, whereas one-electron reduction to the anionic or neutral semiquinone (FAD^•−^/FADH^•^) switches the kinase OFF. The fully reduced form, Aer-FADH_2_, has not been observed *in vitro* but is inferred to promote a kinase ON state (see text).

*E. coli* possesses a repertoire of five dimeric chemoreceptors—Aer, Tsr, Tar, Tap, and Trg—that sense different ligands. Aer responds to changes in oxygen concentration, mediating aerotaxis not by direct interaction with O_2_, but by monitoring the redox state of the electron transport chain (ETC) (18–22). Unlike the other four chemoreceptors, Aer is not an MCP, in that it lacks the E residues important for methylation-based adaptation (23, 24). Also unique is its sensor Per-Arnt-Sim (PAS) domain in the cytoplasm (Fig. 1). PAS binds cofactor flavin adenine dinucleotide (FAD) (23, 25). The HAMP (histidine kinases, adenylyl cyclases, methyl-accepting chemotaxis proteins, and phosphatases) domain plays a crucial role in stabilizing the PAS domain, and the interaction between these domains is vital for the optimal functionality of Aer (26, 27). Redox-dependent conformational changes in the PAS domain alter Aer’s HAMP and kinase interaction domains, modulating CheA activity and thus rotor bias. The differing redox states of FAD, including FAD^2+^ and FADH, when bound to Aer, have been reported to control its signaling output *in vitro* (27, 28). As measured by phosphorylation of CheY, FAD^2+^ activates, and FADH (anionic semiquinone or ASQ) inactivates CheA (28, 29). *In vivo* conditions that alter the cellular redox state, expected to increase FADH_2_ levels, activate CheA as determined by behavioral responses (30–32), although FADH_2_ (hydroquinone or HQ) has not been observed to bind Aer *in vitro* (29) (Fig. 1).

The present study was motivated by our prior observation that inclusion of antibiotics kanamycin or ciprofloxacin in the swarm medium induced a CheY-dependent avoidance or repellent response within a *Serratia marcescens* swarm, an effect not attributable to the antibiotics acting as ligands *per se* (33). Bactericidal antibiotics are known to generate reactive oxygen species (ROS) as secondary consequences of metabolic perturbations involving the tricarboxylic acid cycle and ETC, through damage to iron–sulfur clusters in proteins and via stimulation of the Fenton reaction (34–36). Notably, *Helicobacter pylori* has been shown to swim away from H₂O₂, a known source of ROS (37). Because Aer regulates CheA activity in response to cellular redox changes, we first examined the rotational behavior of individual flagellar motors to monitor the Aer response to ROS and subsequently assessed its response to antibiotics. We find that H₂O₂, a canonical ROS, elicits an immediate chemorepellent response, whereas ciprofloxacin and kanamycin trigger a slightly delayed response. Aer was identified as the primary mediator of these effects, with FAD being essential for its function. These findings broaden the known range of Aer activities to actively avoid oxidizing conditions, including those generated by antibiotics, and provide new assays for monitoring ROS-dependent signaling.

## RESULTS

### *E. coli* responds to H_2_O_2_ as a chemorepellent

**Fig 2:**
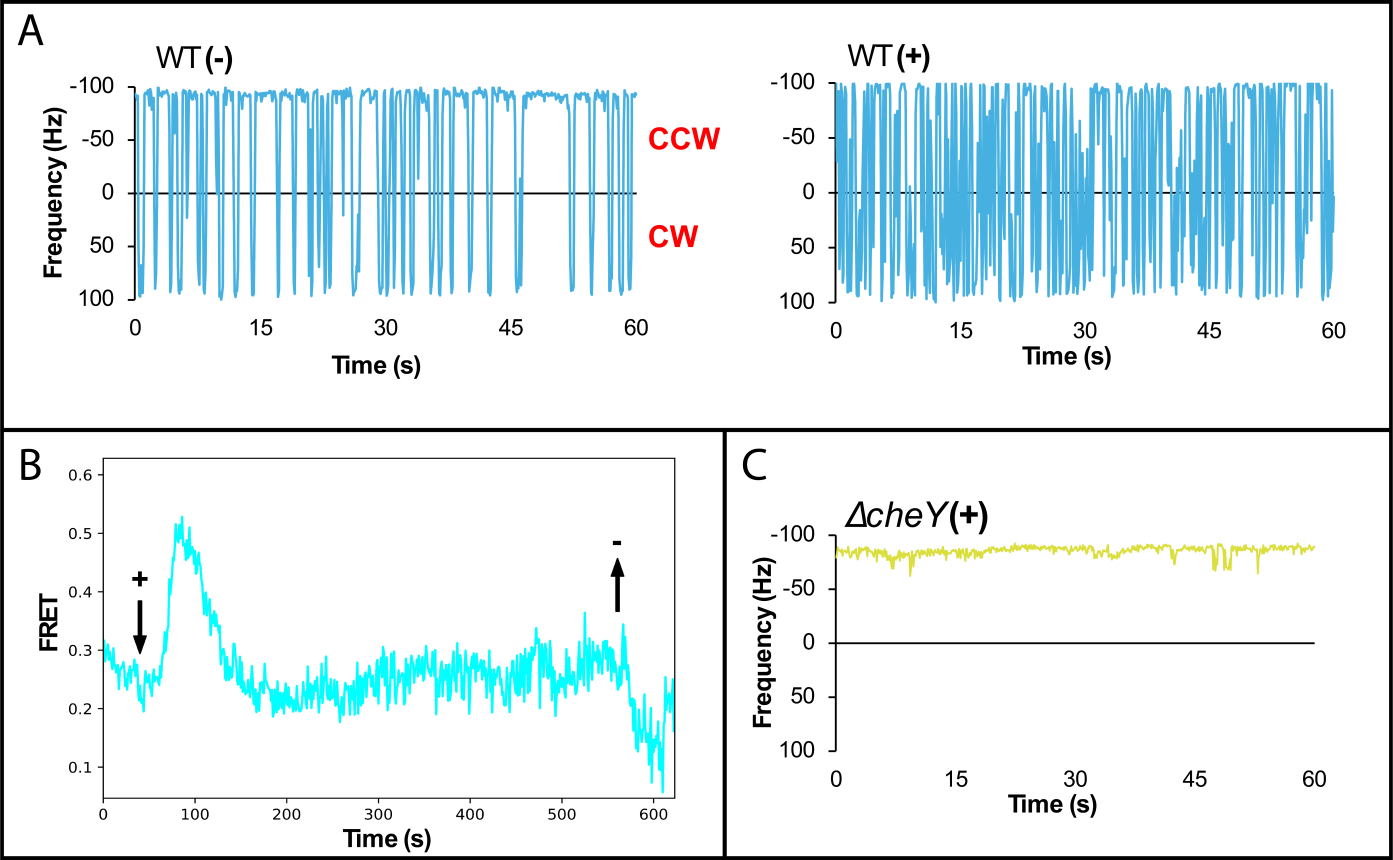
H₂O₂ elicits a repellent response in *E. coli* as measured by two assays. (**A**) Representative motor trace of WT (NBN58) *E. coli* before (−) and immediately after (+) addition of 1 mM H₂O₂. See Fig. S1 for a summary of the cumulative data. (**B**) Förster resonance energy transfer (FRET) measurements in VS115 showing population-level CheA activity (see Materials and Methods). The addition and removal of 1 mM H₂O₂ are indicated by downward (+) and upward arrows (−). The trace is a representative of three independent experiments. (**C**) As in panel A, except with Δ*cheY*.

Since the direction of motor rotation reflects CheA activity, we used the bead assay for motor rotation (see Materials and Methods) to examine the effect of ROS. The *E. coli* strain MG1655 (WT) and its derivatives were used in most assays. Under basal conditions, WT motors exhibited the expected bias, rotating predominantly in the CCW direction with frequent CW reversals (39.07 ± 1.75 reversals per minute or rpm) (38) (Fig. 2A, left). Motor responses to hydrogen peroxide (H₂O₂) were monitored at 1 mM, a concentration previously used in studies reporting antibiotic-induced ROS production (34) and repellent responses to H₂O₂ in *H. pylori* (37). Upon H₂O₂ addition, motor reversals increased significantly (64.53 ± 3.93 rpm), indicative of a repellent response (Fig. 2A, right, and Fig. S1; quantitative data for all motor experiments are shown in the Supplemental material). The motors could not be followed beyond 1 min because the bead invariably detached from the filament stub. Titration experiments showed that as little as 10 µM H₂O₂ was sufficient to elicit a significant repellent response (Fig. S1B).

A complementary assay based on Förster resonance energy transfer (FRET), which monitors CheA activity through the interaction of CheY and CheZ fused to donor and acceptor fluorophores (see Materials and Methods), reproduced the motor response observed in the bead assay: CheA activity increased immediately upon H₂O₂ addition (+) (Fig. 2B). This activity returned to the baseline within approximately 1 min, consistent with adaptation to the chemosensory response, although slower than the adaptation times reported for MCPs (39). The drop in the FRET signal after removal (−) of H₂O₂ resembles that observed following removal of attractant (or repellent) recognized by MCPs, in which the signal often overshoots past the baseline before re-adapting (40), reflecting receptor methylation feedback.

To determine whether the response to H₂O₂ required chemotaxis signaling, we repeated the experiment using a Δ*cheY* mutant, which exhibits an extreme CCW bias due to the absence of the CW signal CheY~P (Fig. 2C). In this strain, H₂O₂ failed to elicit any motor response, confirming that the repellent response to H₂O₂ observed in the WT is mediated through the canonical chemosensory pathway. A summary of motor traces for WT and the *cheY* mutant is shown in Fig. S1A.

### Response to peroxide is via Aer

**Fig 3:**
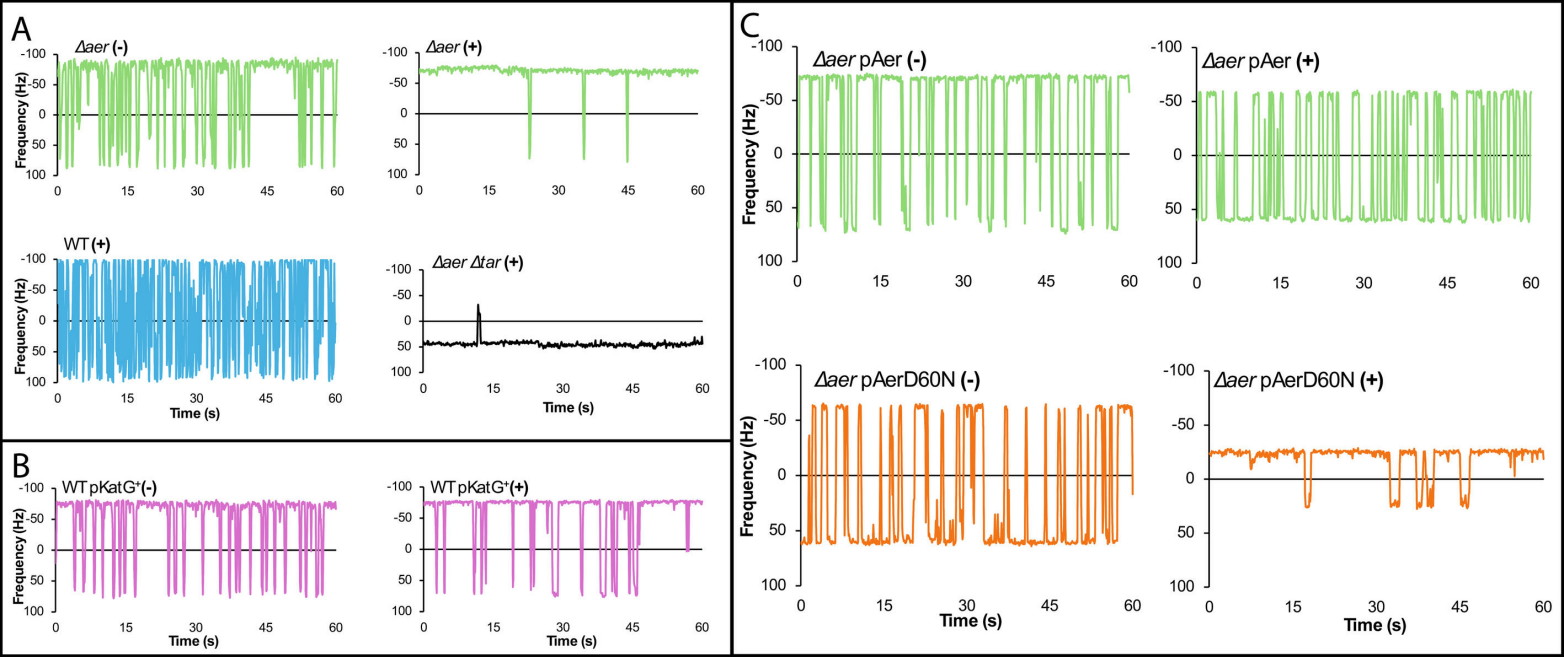
Chemorepellent response to peroxide is via Aer. (**A**) Representative motor traces of WT *E. coli* and its Δ*aer* (NBN30) and Δ*aer* Δ*tar* (NBN130) variants, with (+) and without (−) H₂O₂ addition. Experimental protocol as in Fig. 2A. (**B**) Response to H₂O₂ of WT harboring p*katG* induced (+) with IPTG. (**C**) Comparison of H₂O₂ responses of pAer and pAerD60N introduced into Δ*aer* and induced with IPTG.

Aer is thought to monitor the cellular redox state through its FAD cofactor, with the fully oxidized form activating CheA *in vitro* (28). To test whether Aer plays a similar role *in vivo*, we examined the response of a Δ*aer* strain to H₂O₂. The basal motor bias of Δ*aer* was comparable to that of the WT (Fig. 3A, top left [compare to Fig. 2A left] and Fig. S2A). While WT cells showed a robust repellent response to H₂O₂ (Fig. 3A, bottom left), this response was abolished in the *aer* mutant (Fig. 3A, top right). Interestingly, instead of returning to the basal bias, the mutant now exhibited a clear attractant response.

Because H₂O₂ is a weak acid (pH 6.2 at 1 mM), we considered whether this attractant response might arise from pH sensing by the MCPs Tar and Tsr, known to exhibit opposing responses to pH: Tar senses low pH as an attractant, whereas Tsr mediates a repellent response (41). To test whether Tar contributed to the attractant response in the Δ*aer* mutant, we constructed an *aer tar* double mutant. As expected, this strain displayed a repellent response to low pH, attributable to Tsr (Fig. 3A, bottom right; Fig. S2A).

To determine whether the WT response to H₂O₂ reflected a combined input from Aer responding to ROS and Tsr responding to pH, we conducted two additional tests. First, we introduced into the WT strain a plasmid expressing the catalase–peroxidase KatG, which degrades intracellular H₂O₂ (42). Induction of *katG* with IPTG eliminated the repellent response to H₂O₂ (Fig. 3B; Fig. S2B), indicating that the WT repellent response is due to ROS rather than to pH (note that the motor now shows a more CCW behavior, consistent with Tar sensing low pH as an attractant). Second, because Aer depends on FAD binding to its PAS domain for energy taxis (18), we examined the role of this cofactor using the AerD60N mutant, which is unable to bind FAD (23) and is defective in aerotaxis (24). The *aer* deletion strain complemented with WT Aer (pAer) exhibited the expected repellent response to H₂O₂ (Fig. 3C, top; compare left and right traces). In contrast, the FAD-binding-deficient mutant (pAerD60N) displayed the attractant response characteristic of Tar (Fig. 3C, right traces). Notably, this mutant also exhibited an intrinsic CW bias (Fig. 3C, bottom left), reminiscent of CW-biased, aerotaxis-defective HAMP-domain mutants of Aer (43). These data are summarized in Fig. S2C. We note that Δ*aer* or AerD60N cells exposed to H₂O₂ consistently exhibited lower motor speeds (Fig. S3A and B) (see Discussion).

Together, these findings demonstrate that FAD binding to Aer is essential for eliciting a repellent response to H₂O₂ and establish Aer as an ROS sensor in addition to its known role as an O₂ sensor.

### *E. coli* shows a ROS-dependent repellent response to kanamycin

**Fig 4:**
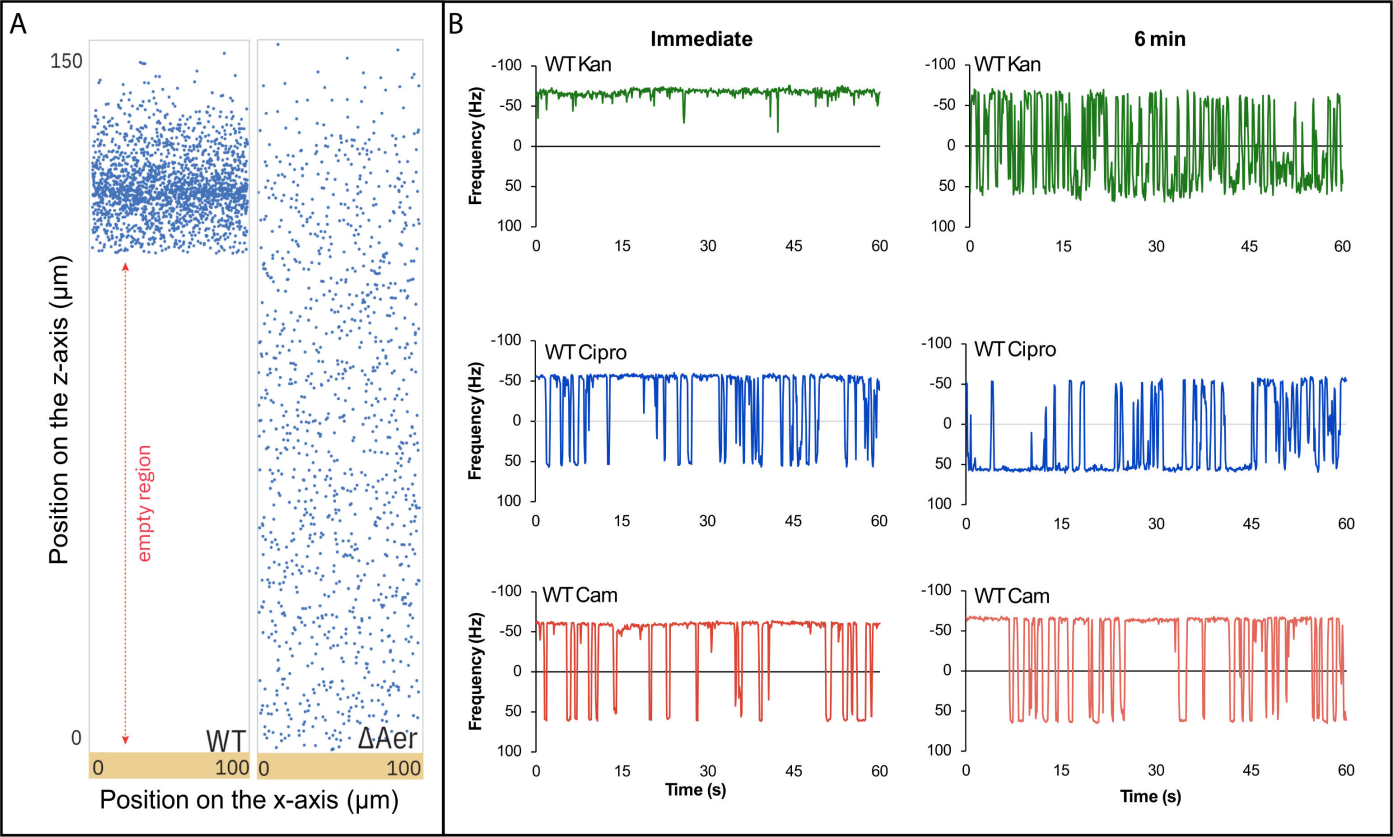
*E. coli* cells show an Aer-dependent avoidance response to kanamycin within a swarm. (**A**) *z*-axis tracking of GFP-labeled cells in WT (NBN66) and *Δaer* (NBN67) swarms grown on medium containing 3 µg/mL kanamycin, as described in Materials and Methods. The *z*-axis indicates the vertical position within the colony (total height ≈ 150 µm), and the *x*-axis indicates the horizontal position of each tracked cell. “Empty region” denotes the absence of GFP-labeled cells. Data were collected from >10 experiments, yielding ~1,000 points per condition. Statistical significance was calculated relative to the no-treatment control using a paired Student's *t*-test (**P < 0.01; ***P < 0.001). (**B**) Representative motor traces of WT *E. coli* treated with kanamycin (10 µg/mL), ciprofloxacin (1 µg/mL), and chloramphenicol (15 µg/mL). Motors were tracked continuously for 10 min following antibiotic addition.

Having established that *E. coli* exhibits a chemosensory response to ROS via Aer, we next tested whether *E. coli* swarms display the avoidance response to sublethal levels of kanamycin and ciprofloxacin previously observed in *S. marcescens* swarms (33). Using defocused epifluorescence video imaging, in which the z-position of GFP-labeled cells within the swarm colony is inferred from the diameter of their diffraction ring (33), we compared the distribution of GFP-labeled WT cells and their Δ*aer* variant grown on swarm medium containing sublethal kanamycin (3 µg/mL) (see Materials and Methods). The average colony height at the observed region in the swarm was approximately 150 µm. When viewed from above, GFP-cells located near the agar surface appear in focus, whereas cells positioned higher in the colony display a fluorescent diffraction ring whose diameter increases with the distance from the bottom surface. WT *E. coli* cells preferentially localized away from the agar surface, whereas the *aer* mutant cells were uniformly distributed throughout the colony (Fig. 4A). These results with *E. coli* mirror those reported for *S. marcescens* swarms (33), identifying in addition that Aer is responsible for the avoidance response.

Since bactericidal antibiotics are known to induce ROS in bacteria, we next monitored the motor responses of *E. coli* cells to sublethal concentrations of kanamycin and ciprofloxacin—antibiotics reported to generate ROS—and to chloramphenicol, a bacteriostatic antibiotic that reportedly does not (34). Because antibiotics generate ROS as a secondary effect of their primary targets, we did not expect an immediate response as seen with exogenous H₂O₂. Accordingly, motor rotation was tracked for 10 min following antibiotic addition in WT cells. Kanamycin elicited a biphasic response: an immediate attractant phase (Fig. 4B, top panel, left), followed by a delayed repellent phase beginning at approximately 6 min (Fig. 4B, top panel, right). Ciprofloxacin induced a similar delayed repellent response but lacked the initial attractant phase (Fig. 4B, middle panel). In contrast, chloramphenicol elicited no detectable response within this time window (Fig. 4B, bottom panel).

**Fig 5:**
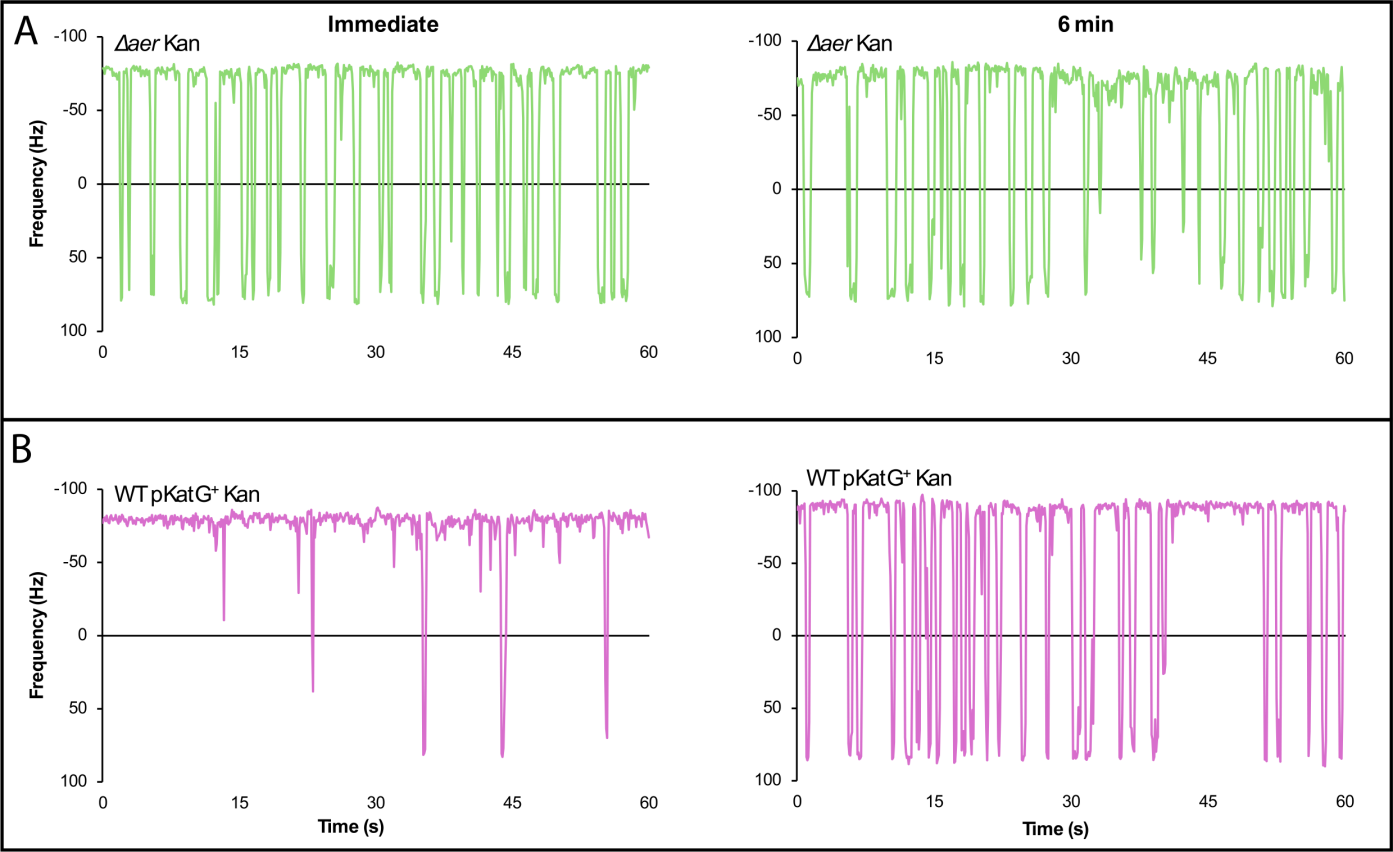
Repellent antibiotic response is Aer and ROS-dependent. Representative motor traces of (**A**) Δ*aer* (NBN 30) and (**B**) WT harboring p*katG* (NBN 33) treated with kanamycin (10 µg/mL). Motors were tracked continuously for 10 min after the addition of the antibiotic. Other labels, as in Fig. 4.

To determine whether the repellent response to kanamycin is Aer- and ROS-dependent, we repeated the experiment using a Δ*aer* mutant, as well as in WT cells expressing the catalase–peroxidase KatG. The *aer* mutant failed to exhibit either the early attractant or late repellent response (Fig. 5A, top). In WT cells expressing KatG, the delayed repellent phase was abolished, whereas the initial attractant phase persisted (Fig. 5B, bottom). These results confirm that the antibiotic-induced chemorepellent response is mediated through Aer and depends on ROS signaling. A summary of the data presented in Fig. 5 is shown in Fig. S4.

## DISCUSSION

The results presented in this study demonstrate that the chemoreceptor Aer is employed by *E. coli* to actively avoid antibiotics. Using two complementary assays—motor behavior and FRET—we show that Aer, previously known to mediate positive chemotaxis toward O₂, also mediates negative chemotaxis in response to ROS (Fig. 2 and 3). Together, these assays provide a sensitive and convenient means to monitor intracellular ROS. Leveraging Aer’s redox sensitivity in this way, our findings support the growing consensus that bactericidal antibiotics, in addition to their primary targets, kill cells in part through ROS generated by downstream metabolic reactions (36).

At the molecular level, the negative chemotaxis response to ROS observed here is consistent with *in vitro* studies demonstrating that Aer bound to reduced FADH signals an attractant response, whereas the oxidized FAD form signals repulsion (28). *In vivo* exposure to H₂O₂ is therefore expected to shift the intracellular flavin pool toward the oxidized state, in agreement with our behavioral data. These results are also consistent with earlier observations that pure O₂, as well as the addition of quinones, induces tumbling in *E. coli* (19, 32). In contrast, partially oxidizing conditions—achieved by adding 1%–20% O₂ after cells have been maintained in a reducing environment—elicit smooth swimming (25). Together, these findings support the current model in which Aer predominantly exists under steady-state conditions in the ASQ state, promoting CheA-OFF (CCW) behavior (29), and where either oxidation or reduction of the ASQ shifts Aer to a CheA-ON state, increasing CW rotation and tumbling (22, 29) (Fig. 1). Notably, the reduced HQ form of Aer (FADH_2_) has not been observed *in vitro* (28, 29), leading to the speculation that under strongly reducing conditions, flavin may dissociate from Aer. In this model, the CW-activating “reduced” signaling state of Aer may correspond to an apo form of the protein (29). This idea is supported by the observation that many Aer mutations that constitutively activate signaling are predicted to disrupt flavin binding (43).

From a systems perspective, Aer is distinct among the five *E. coli* chemoreceptors: it is expressed at less than 10% of the level of the major MCPs Tsr and Tar, whose estimated copy numbers range from 10,000 to 30,000 per cell (44), and it lacks the methylation-based adaptation mechanism used by these receptors (18, 23). Despite this, Aer adapts to O₂ stimuli in soft agar assays (24). Using FRET, we show that Aer can also adapt to ROS signals, although more slowly than MCPs (Fig. 2B). Future FRET measurements using Aer as the sole receptor alone will be important for determining the extent to which adaptation arises from Aer itself versus contributions from MCP signaling networks.

**Fig 6:**
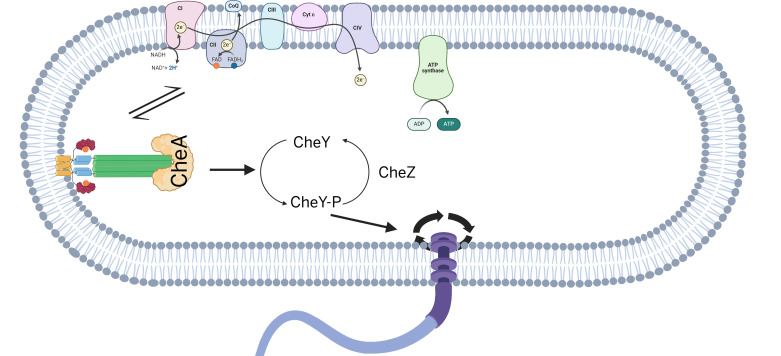
Summary. Aer-bound FAD/FADH reflects global electron transport chain activity by equilibrating with the cytoplasmic redox state in *E. coli*, mediating both positive chemotaxis toward O₂ and negative chemotaxis to ROS.

One unexpected observation was that motor speeds are reduced in Δ*aer* cells exposed to H₂O₂ (Fig. S3A and B). Although direct measurements of Aer copy number are not available, its abundance is expected to be low (44), making it unlikely that Aer deletion substantially elevates cytoplasmic FAD levels. However, Aer expression has been reported to be linked to cellular flavin levels in a poorly understood manner (18, 45), raising the possibility that indirect physiological effects on energy metabolism could reduce the PMF available to power the flagellar motor. Figure 6 summarizes our current understanding of Aer function as advanced in this study.

We also replicated in *E. coli* the avoidance responses to kanamycin and ciprofloxacin previously observed in *S. marcescens* swarms (33), and further demonstrated that the response to kanamycin is Aer-dependent (Fig. 4A). Although the contribution of ROS in antibiotic lethality was once debated (46), accumulating evidence now supports a model in which bactericidal antibiotics accelerate cellular metabolism, leading to downstream ROS generation that contributes to cell death (36). Traditionally, ROS production following antibiotic treatment has been monitored using genetically encoded sensors (47), oxidation-sensitive dyes (34, 48), or oxygen consumption assays (49), which typically report changes over tens of minutes. In contrast, Aer-mediated CheA activation in response to H₂O₂ occurred immediately in both bead and FRET assays (Fig. 2), and avoidance responses to sublethal concentrations of bactericidal antibiotics were detected within minutes (Fig. 4B). These observations indicate that Aer provides a rapid and sensitive *in vivo* readout of ROS generation. Notably, no ROS-dependent response was detected following exposure to the bacteriostatic antibiotic chloramphenicol within the 10-min observation window (Fig. 4B), consistent with its distinct mode of action.

The biphasic motor response to kanamycin—an initial attractant phase, followed by a delayed repellent phase (Fig. 4B), with only the latter abolished by KatG expression (Fig. 5B)—highlights the dynamic nature of Aer-mediated signaling. Aminoglycoside entry is known to disrupt PMF (50), and lowering PMF is perceived as a higher load on the motor, which produces a CCW bias (51–53), providing a possible explanation for the initial attractant response to kanamycin. That this response disappears when Aer is deleted (Fig. 5A) might suggest that Aer plays a more important role in PMF sensing than previously thought. The differences in the motor responses of the three structurally different antibiotics tested reveal differences in their modes of entry and in cellular redox.

The role of Aer in ROS sensing parallels that of *H. pylori*’s TlpD, a cytoplasmic chemoreceptor that mediates repulsion from oxidative stress to help the bacterium evade host-derived ROS (37). TlpD has no identifiable domains that would bind FAD/FADH, but possesses a CZB domain at its C terminus, which binds zinc via histidine and cysteine residues (54). Other ROS-sensing proteins in both prokaryotes and eukaryotes use cysteine as a way to sense redox status (55), so this mechanism is speculated to operate in TlpD as well (54). Together, these findings provide insight into how bacteria might evade ROS in their natural environments, whether host-derived or antibiotic-generated. By actively moving away, bacteria may reduce local drug exposure and oxidative damage, adding a behavioral dimension to antibiotic resistance strategies (Fig. 4A). Targeting chemotaxis pathways—or Aer signaling specifically—could therefore enhance bacterial susceptibility to ROS-generating antibiotics and limit their capacity to evade treatment.

## MATERIALS AND METHODS

### Strains and growth conditions

All strains and plasmids used in this study are listed in Tables S1 and S2. *E. coli* cultures were grown in Lennox Broth (LB; 20 g/L, Fisher BioReagents). Antibiotics were added at the following concentrations for strain selection: ampicillin (100 µg/mL), chloramphenicol (20 µg/mL), and kanamycin (50 µg/mL). For plasmid induction, 50 µM isopropyl-β-D-thiogalactopyranoside (IPTG) or 0.2% L-arabinose was used, unless otherwise indicated. Cell growth was monitored by measuring optical density at 600 nm (OD₆₀₀) using a spectrophotometer.

All FRET experiments were performed with RP437, a derivative of *E. coli* K-12 harboring CheYZ and *fliC* mutations and transformed with two plasmids: pSJAB106, from which CheZ-YFP and CheY-mRFP1 are expressed in tandem on an IPTG-inducible promoter, and pZR1, from which “sticky” FliC^∗^ is expressed on a sodium-salicylate (NaSal) inducible promoter. Cells were grown overnight in TB broth (1% bacto-tryptone, 0.5% NaCl), then diluted 1:100 into 10 mL of fresh TB supplemented with 50 µM IPTG to induce the FRET pair, and 3 µM NaSal to induce sticky FliC^∗^ for cell adhesion to glass coverslips, with 100 µg/mL ampicillin and 34 µg/mL chloramphenicol for plasmid retention.

### Mutagenesis and plasmid constructions

The WT parent strains of *E. coli* used were MG1655. Mutant strains were generated by inserting a kanamycin resistance (KAN) cassette into the target gene using the Keio collection (56). Mutations were transferred to fresh strain backgrounds by P1 Cm transduction, and KAN cassettes were excised via FLP recombinase expression from pCP20 (57). All resulting strains were verified by DNA sequencing.

To construct plasmids of interest, specific genes were amplified from the wild-type (WT) strain and cloned into pBAD33 via Gibson Assembly (58). Site-directed mutagenesis of *aer* was achieved using primers containing base substitutions to amplify the entire pNBN4 plasmid. For inducible expression of KatG, we used the ASKA library plasmid (ID: JW3914) containing the corresponding open reading frame on pCA24N (59).

### Bead assay

Bead assays were performed as previously described (38, 60). *E. coli* Δ*fliC* strains were complemented with plasmid pFD313 expressing sticky FliC^∗^ under a constitutive promoter (61). Flagella were sheared by passing cells through two syringes connected by a 7-inch polyethylene capillary. A 40 µL cell suspension was applied to a poly-L-lysine-coated coverslip attached to the glass slide with double-sided tape, incubated for 10 min, and washed with motility buffer to remove unattached cells (62). Subsequently, 40 µL of a 1:50 dilution of polystyrene beads was added, incubated for 10 min, and excess beads were washed away.

Bead rotation was recorded at 1,000 frames/s using a CCD camera (ICL-B0620M-KC0; Imperx, Boca Raton, FL). Videos were analyzed with custom LabVIEW 2012 software as previously described (60).

### Ligand exposure and chemotactic response recording

Once a properly attached bead on a flagellar stub was identified within the microscope’s field of view, a 60-s baseline recording was obtained. Ligands were then introduced through the top of the chamber and gently drawn out from the bottom using a Kimwipe to maintain smooth flow. Post-exposure recordings were collected for 60 s for peroxide treatments and up to 10 min for antibiotic treatments, or until the bead detached from the flagellum. The concentrations of the antibiotics tested were 10 µg/mL kanamycin, 1 µg/mL ciprofloxacin, and 15 µg/mL chloramphenicol. For hydrogen peroxide, the concentrations tested were 1 mM, 100 µM, 50 µM, and 10 µM.

### Data acquisition and analysis

Bead assay data included both rotation frequency and direction for individual motors. CCW rotations were assigned negative values, while CW rotations were positive. CW bias, defined as the fraction of time a motor rotated CW within a given interval, was calculated as the ratio of positive to total data points in that interval (63, 64).


$$CW_{bias}=\;\frac{CW_{time}}{CW_{time}\;+\;CCW_{time}}.$$

Batch processing was performed using a custom Python script (all the Python code for this work is available at https://github.com/nbnnpl01/CW-bias-calculation.git). For long recordings, CW bias was smoothed using a 30 s running average. Statistical significance was determined using Student’s *t*-test comparing each sample to its corresponding WT control.

### Three-dimensional tracking of *E. coli* cells within swarming colonies

Cells were grown on swarm plates containing 2.5 g/L LB, 0.45% agar, and 0.5% glucose, supplemented with 3 µg/mL kanamycin. GFP-labeled cells (pJP10120) were mixed 1:100 with unlabeled cells prior to inoculation. A 20 µL inoculum was placed at the center of the plate, and colonies were grown at 30°C and 95% relative humidity for 9 h. Wild-type and Δ*aer* swarms of *E. coli* were grown and monitored separately but under identical conditions.

Swarm colonies (~4 cm diameter) were harvested, and imaging was performed in the colony interior, ~0.5 cm from the colony edge, at a height of ~150 µm. Cell Z-positions were determined using an off-focus diffraction ring method (33), where the ring diameter correlates with cell height (z-resolution ~0.5 µm). Data were collected from multiple experiments (≈1,000 points per data set) using a Zeiss Axio Imager Z2 microscope with a 63× objective (field of view = 100 µm × 100 µm) and a Neo Andor camera. Filter set 46 (YFP) was used instead of set 38 (GFP) to avoid light-induced pausing.

### *In vivo* single-cell FRET microscopy

Single-cell FRET imaging and sample preparation were performed as described previously (65). Cells were grown and washed as outlined above. Imaging was conducted using a custom microfluidic device designed for single-cell FRET experiments. The device contained seven inlets for delivering motility media (10 mM potassium phosphate/0.1 mM EDTA/1 μM methionine/10 mM lactic acid, pH 7) (66) with varying ligand concentrations, two side outlets, and one main outlet. Prior to each experiment, cells were introduced through the main outlet, where they circulated through the main chamber and exited through one of the side outlets, while the other side outlet remained unused. After loading, both side outlets were sealed to redirect flow toward the main outlet for imaging.

Imaging was performed on a Nikon Eclipse Ti-E inverted microscope equipped with a 60× oil-immersion TIRF objective (CFI Apo TIRF 60× Oil, Nikon). Yellow fluorescent proteins were excited using a SOLA SE LED light source (Lumencore) through excitation filters (59026x, Chroma; FF01-500/24-25, Semrock) and a dichroic mirror (FF520-Di02, Semrock). Emission signals were separated into donor and acceptor channels using an OptoSplit II system (Cairn) and collected through emission filters (FF01-542/27 and FF02-641/75, Semrock) on an ORCA-Flash 4.0 V2 camera (Hamamatsu). mRFP1 imaging was performed similarly, using an excitation filter FF01-575/05-25 and a dichroic mirror FF593-Di03-25×36. All images were acquired with an exposure time of 50 ms.

### FRET data analysis

Single-cell fluorescence time series were analyzed as previously described (65, 67, 68). Cells were segmented, and donor (D) and acceptor (A) signals were extracted using in-house software. Photobleaching was corrected by fitting donor and acceptor traces to bi-exponential decay functions.

To calculate FRET from fluorescence time series, we used the E-FRET method (67). The E-FRET index is given by


$$\text{FRET}=E_{app}=\frac{I_{DA}-aI_{AA}-dI_{DD}}{I_{DA}-aI_{AA}+\left(G-d\right)I_{DD}},$$

where $I_{DA}$ is FRET-acceptor emission intensity from donor excitation, $I_{AA}$ is acceptor emission with acceptor excitation, $I_{DD}$ is donor emission from donor excitation, with a,G,d being optical constants that depend on the FRET pair and optical setup which were determined by an independent experiment with strains that express only CheY-mRFP or only CheZ-mYFP.

FRET time series were normalized for each cell using the minimum and maximum FRET values measured during saturating stimuli at the start and end of experiments to estimate kinase activity.

## Data Availability

All data supporting the findings of this study are available within the article and its supplemental material.

## SUPPLEMENTAL MATERIAL

Supplemental material mbio.00381-26-s0001.docx Supplemental figures and tables.

ASM does not own the copyrights to Supplemental Material that may be linked to, or accessed through, an article. The authors have granted ASM a non-exclusive, world-wide license to publish the Supplemental Material files. Please contact the corresponding author directly for reuse.
