# Supplementary material for: Aer is a bidirectional redox sensor mediating negative chemotaxis to antibiotic-induced ROS in Escherichia coli
Source: mBio. 2026 Mar 23;17(4):e00381-26. doi: 10.1128/mbio.00381-26 (PMC13059710; doi:10.1128/mbio.00381-26)
Supplement: Supplemental material — Supplemental figures and tables. [file mbio.00381-26-s0001.docx]

**Supplementary Figures and Tables**

**Figures**


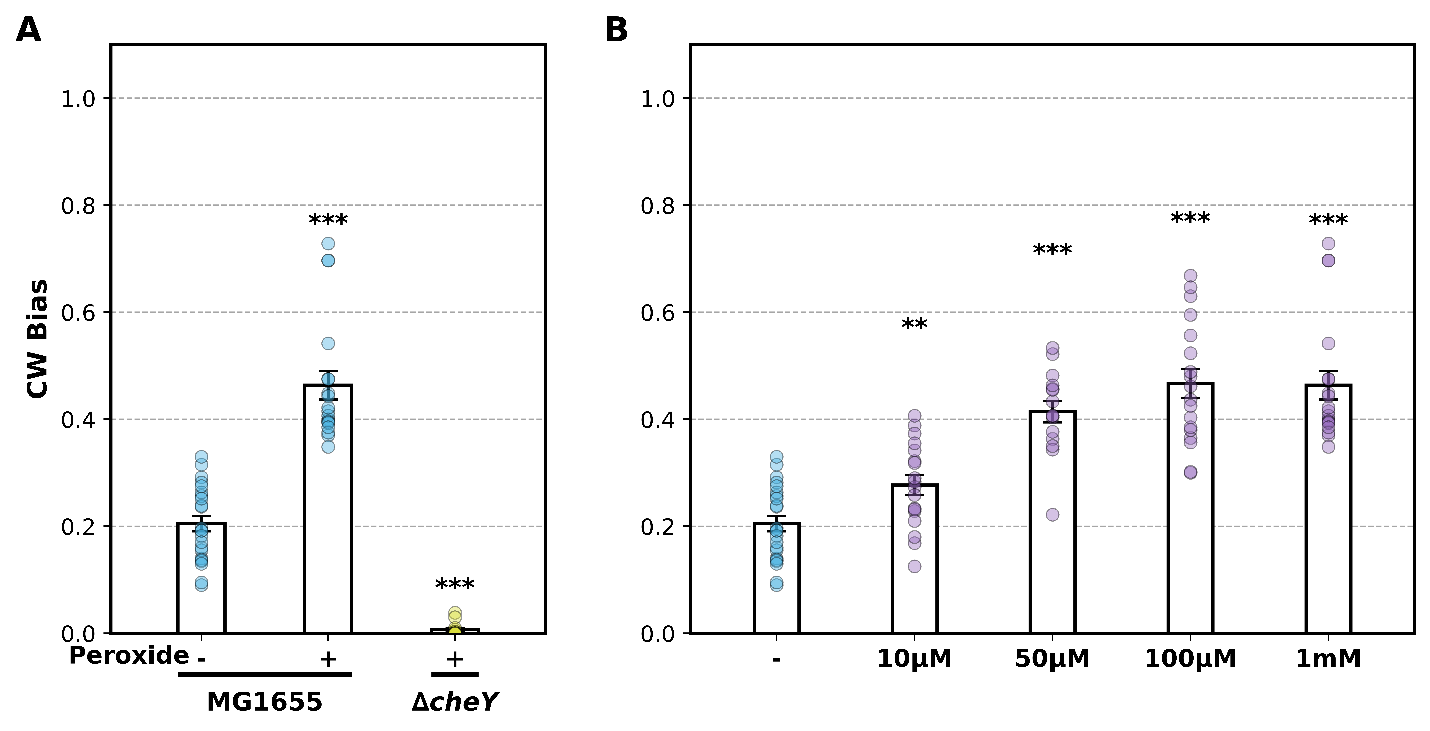


**Fig. S1**. Summary of chemosensory response to H₂O₂ expressed as CW bias. At least 15 individual motors were measured for each data set. CW bias calculation is described in Methods. (A) Representative traces of these data sets are shown in Fig. 1A and C. (B) Titration of H₂O₂ to determine minimal concentration required to elicit a CW response in WT. Statistical significance was calculated against the no-treatment control using paired students t-test (**p<0.01; ***p<0.001).


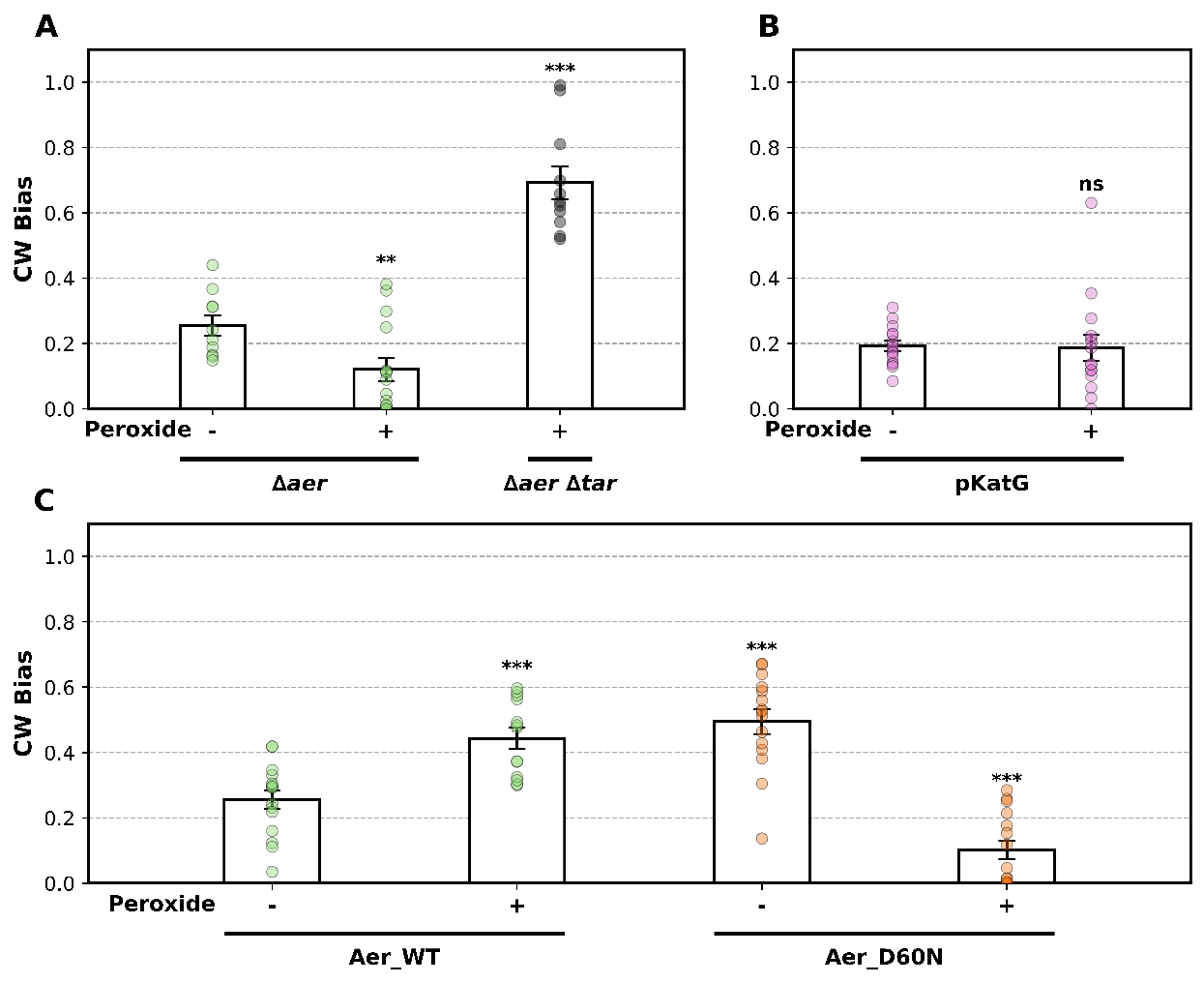


**Fig. S2**. Summary of chemosensory response of Δ*aer,* Δ*aer* Δ*tar* and *aer*(D60N) to H₂O₂. At least 15 individual motors were measured for each data set, plotted CW bias. Representative traces of these data sets are shown in Fig. 3.


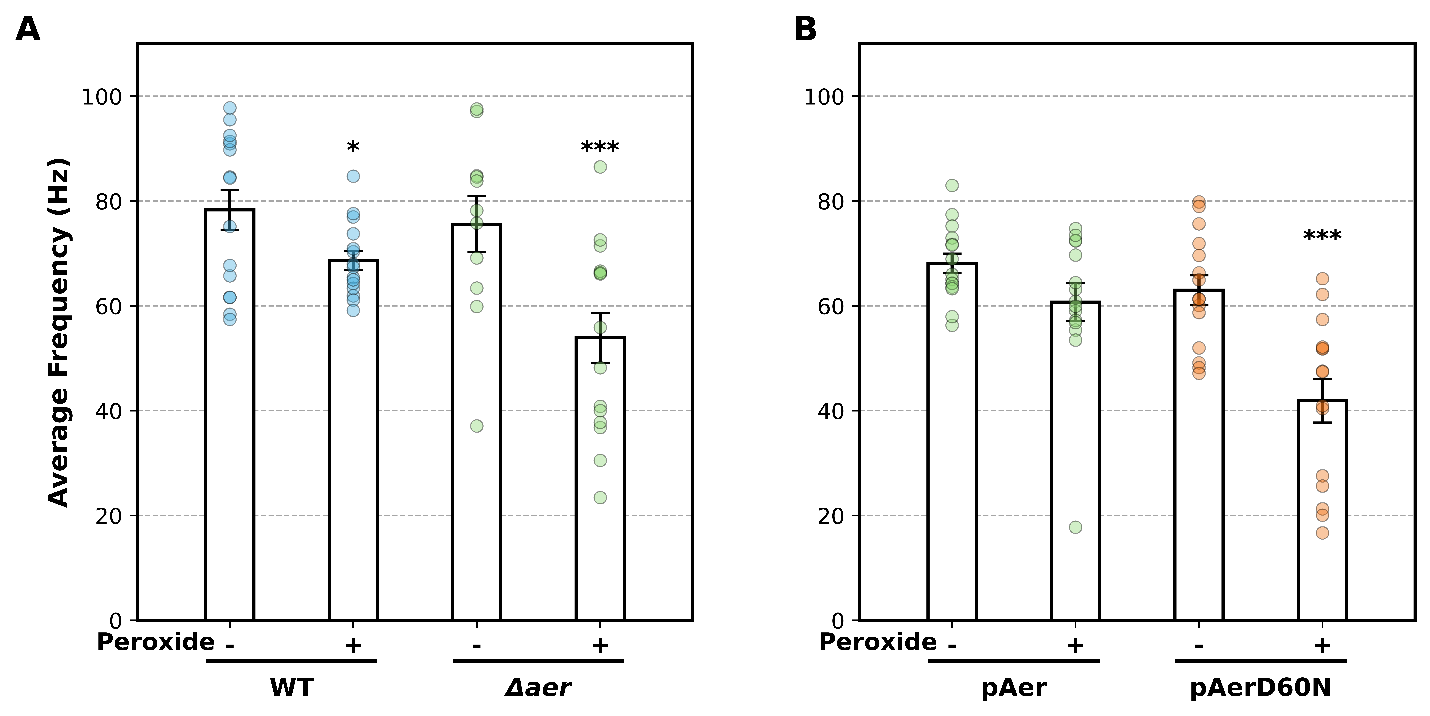


**Fig. S3**. Lower motor speeds in Δ*aer* exposed to H₂O₂. Representative traces for the response of Δ*aer* and Δ*aer* pAerD60N are shown in Figs. 3A and C.


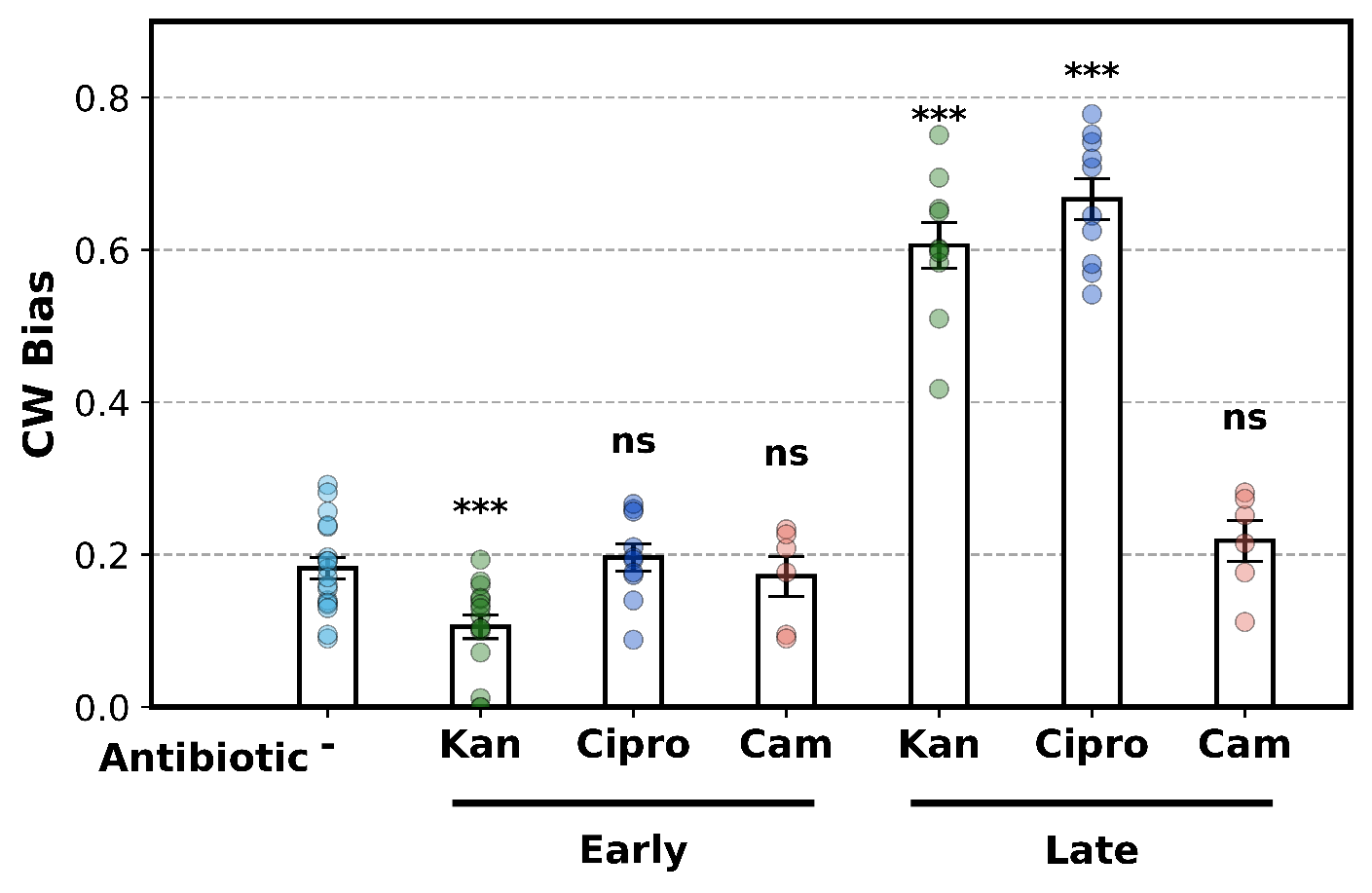


**Fig. S4.** Summary of WT chemosensory response to kanamycin, ciprofloxaxcin, chloramphenicol. 15 individual motors measured for each data set, plotted CW bias. See Figs 4B and 5A for other descriptions.

**Tables**

**Table S1: Strains**

| **Strain** | **Genotype/Description** | **Reference** |
| --- | --- | --- |
| MG1655 | Wild type *E. coli* F^-^ λ^-^ *ilvG*- *rfb*-50 *rph*-1 | Lab collection |
| NBN28 | MG1655 ∆*fliC* | This work |
| NBN58 | NBN28 + pFD313 | This work |
| NBN64 | MG1655 *∆aer* | This work |
| NBN66 | MG1655 + pJP1020 | This work |
| NBN67 | NBN64 + pJP1020 | This work |
| NBN69 | NBN28 *∆cheY* | This work |
| NBN70 | NBN69 + pFD313 | This work |
| NBN30 | NBN28 *∆aer* + pFD313 | This work |
| NBN31 | NBN30 + pNBN4 | This work |
| NBN32 | NBN30 + pNBN5 | This work |
| NBN33 | NBN58 + pCA24N | This work |
| NBN130 | NBN28 *∆aer* *∆tar* + pFD313 | This work |
| VS115 | RP437 ∆*cheY* ∆*cheZ* ∆*fliC +* pSJAB106 | (1) |

**Table S2: Plasmids**

| **Plasmid** | **Expressed Protein** | **Host Plasmid** | **Resistance** | **Induction** | **Reference** |
| --- | --- | --- | --- | --- | --- |
| pCP20 | FLP recombinase | **N.A** | Ampicillin | Constitutive | (2) |
| pFD313 | FliC^sticky^ | pTrc99a | Ampicillin | IPTG | (3) |
| pCA24N | KatG | **N.A** | Chloramphenicol | IPTG | (4) |
| pNBN4 | Aer | pBAD33 | Chloramphenicol | Arabinose | This work |
| pNBN5 | AerD60N | pBAD33 | Chloramphenicol | Arabinose | This work |
| pJP1020 | GFP | pTrc99a | Ampicillin | IPTG | (5) |
| pSJAB106 | CheZ-5G-YFP/CheY-5G-mRFP1 | pTrc99a | Ampicillin | IPTG | (6) |
| pZR 1 | FliC^sticky^ | pKG116 | Chloramphenicol | NaSal | (6) |

**N.A**: Not applicable

**References**

1. K. Kamino, J. M. Keegstra, J. Long, T. Emonet, T. S. Shimizu, Adaptive tuning of cell sensory diversity without changes in gene expression. *Sci Adv* **6** (2020).

2. K. A. Datsenko, B. L. Wanner, One-step inactivation of chromosomal genes in *Escherichia coli* K-12 using PCR products. *Proc Natl Acad Sci U S A* **97**, 6640-6645 (2000).

3. G. Kuwajima, Construction of a minimum-size functional flagellin of Escherichia coli. *J Bacteriol* **170**, 3305-3309 (1988).

4. M. Kitagawa *et al.*, Complete set of ORF clones of Escherichia coli ASKA library (a complete set of E. coli K-12 ORF archive): unique resources for biological research. *DNA Res* **12**, 291-299 (2005).

5. G. Ariel *et al.*, Swarming bacteria migrate by Levy Walk. *Nat Commun* **6** (2015).

6. J. M. Keegstra *et al.*, Phenotypic diversity and temporal variability in a bacterial signaling network revealed by single-cell FRET. *Elife* **6** (2017).
